# Cytoplasmic Polyadenylation Element Binding Proteins CPEB1 and CPEB3 Regulate the Translation of FosB and Are Required for Maintaining Addiction-Like Behaviors Induced by Cocaine

**DOI:** 10.3389/fncel.2020.00207

**Published:** 2020-07-09

**Authors:** Bettina Drisaldi, Luca Colnaghi, Amir Levine, YanYou Huang, Anna M. Snyder, Daniel J. Metzger, Martin Theis, Denise B. Kandel, Eric R. Kandel, Luana Fioriti

**Affiliations:** ^1^Department of Neuroscience, Columbia University, New York, NY, United States; ^2^Mailman School of Public Health, Columbia University, New York, NY, United States; ^3^Department of Epidemiology of Substance Abuse, New York State Psychiatric Institute, New York, NY, United States; ^4^Kavli Institute for Brain Science, Columbia University, New York, NY, United States; ^5^Mortimer B. Zuckerman Mind Brain Behavior Institute, Columbia University, New York, NY, United States; ^6^Howard Hughes Medical Institute, Chevy Chase, MD, United States; ^7^Dulbecco Telethon Institute, Istituto di Ricerche Farmacologiche Mario Negri, Milan, Italy

**Keywords:** cytoplasmic polyadenylation, protein translation, cocaine, addictive behavior, delta FosB, FosB, long-term memory (LTM)

## Abstract

A recurrent and devastating feature of addiction to a drug of abuse is its persistence, which is mediated by maladaptive long-term memories of the highly pleasurable experience initially associated with the consumption of the drug. We have recently found that members of the CPEB family of proteins (Cytoplasmic Polyadenylation Element-Binding Proteins) are involved in the maintenance of spatial memory. However, their possible role in the maintenance of memories that sustain addictive behavior has yet to be explored. Little is known about any of the mechanisms for maintaining memories for addictive behavior. To address the mechanisms whereby addictive behavior is maintained over time, we utilized a conditional transgenic mouse model expressing a dominant-negative version of CPEB1 that abolishes the activity in the forebrain of two of the four CPEB isoforms (CPEB1 and CPEB3). We found that, following cocaine administration, these dominant-negative (DN) CPEB mice showed a significant decrease, when compared to wild type (WT) mice, in both locomotor sensitizations and conditioned place preference (CPP), two indices of addictive behavior. Supporting these behavioral results, we also found a difference between WT and DN-CPEB1-3 mice in the cocaine-induced synaptic depression in the core of the Nucleus Accumbens (NAc). Finally, we found that (1) CPEB is reduced in transgenic mice following cocaine injections and that (2) FosB, known for its contribution to establishing the addictive phenotype, when its expression in the striatum is increased by drug administration, is a novel target of CPEBs molecules. Thus, our study highlights how CPEB1 and CPEB3 act on target mRNAs to build the neuroadaptative implicit memory responses that lead to the development of the cocaine addictive phenotypes in mammals.

## Introduction

Drug addiction carries enormous societal and clinical burdens due in large part to the persistence of the negative consequences of addictive behaviors. Despite many available treatments for withdrawal from drugs of abuse, individuals who succeed in stopping the use of drugs are at risk for relapse for periods ranging from days to decades. Addiction is thought to be caused, in part, by powerful and long-lasting memories of the drug experience. These memories are thought to be responsible for the relapses that occur upon exposure to drug cues (Hyman et al., 2006). Indeed, several studies have found that addiction and long-term memory share several molecular mechanisms (Robbins and Fioriti, 1963; Nestler and Malenka, 2004; Hyman et al., 2006). In both cases, visual cues play an important role in recalling memories. In drug addiction, cues associated with drug consumption, such as people or places, can lead to the recall of old memories that trigger the urge for drug use (Kalivas and Volkow, 2005). In contrast to spatial memories, where the hippocampus plays a central role, compulsive drug seeking and long-term relapse vulnerability are thought to result from drug-induced neuroadaptations in the nucleus accumbens (NAc) and in both the mesocorticolimbic dopamine and the glutamatergic corticolimbic circuitries, in which the dopamine projections are embedded (Wolf, 1998; Nestler, 2001; Ungless et al., 2001; Nestler and Malenka, 2004; Kalivas and Volkow, 2005).

Most of the research that has been carried out on the molecular mechanisms of learning and memory as well as on synaptic plasticity implicated in drug addiction, has focused on the regulation of transcription. However, it is now clear that changes in gene expression, which result in long-lasting alteration of synaptic proteins, and the remodeling and growth of synaptic connections, also require the regulation of local translation at the activated synapses. This is needed to trigger the synthesis of new proteins required for the stabilization of the new synaptic growth (Kandel, 2001; Kandel et al., 2014). For example, the translational regulator *Aplysia*-Cytoplasmic Polyadenylation Element-Binding (CPEB) maintains local protein synthesis of its mRNA targets at the synapses that contribute to long-term facilitation (Si et al., 2003a,b). To regulate translation, soluble monomers of Aplysia-CPEB bind to a six-nucleotide specific sequence, the cytoplasmic polyadenylation element (CPE), which is located in the 3′ untranslated region (Russo et al., 2008) of target mRNAs (Hake and Richter, 1994). This binding by CPEB monomers represses the translation of mRNAs. But, upon synaptic activation induced by learning, CPEB is activated to form aggregates that constitute the active form of CPEB capable of promoting the polyadenylation of target mRNAs and their translation into proteins that sustain synaptic growth and memory storage (Wu et al., 1998; Si et al., 2003a, 2010; Alarcon et al., 2004; Atkins et al., 2004; Du and Richter, 2005; Berger-Sweeney et al., 2006).

In mammals, four isoforms of CPEB—CPEB1-4—have been found (Theis et al., 2003) and have been implicated in a variety of biological contexts: from translational regulation of embryonic cell division (Groisman et al., 2000) to hippocampal synaptic plasticity and long-term memory (Fioriti et al., 2015). To better understand the role of mammalian CPEB proteins in mediating long-term memories in the hippocampus, we generated a conditional transgenic mouse [dominant-negative (DN)-CPEB] by expressing a dominant-negative inhibitor of CPEB. In the forebrain, the DN-CPEB mice express a truncated version of CPEB1 lacking its N-terminal region, resulting in a gene product that can bind to the CPE of target mRNAs of CPEB1 but cannot induce translation of the messengers, therefore acting as a dominant-negative inhibitor. Since CPEB3 mRNA is a direct target of CPEB1, the DN-CPEB mice also have reduced levels of CPEB3. Consistent with these findings, the DN-CPEB mice exhibit significant deficiencies in the maintenance of the late phase of LTP and in the ability to maintain spatial long-term memory. A mouse with an exclusive conditional knock out of CPEB3 recently created in our laboratory (Fioriti et al., 2015) has confirmed that selective absence of CPEB3 alone induces deficits in hippocampal long-term memory.

We here ask whether addiction-associated phenotypes are also maintained by the activation and function of CPEB1 and CPEB3 in the striatum. We found that the DN-CPEB mice exhibit three characteristic features: (1) markedly reduced sensitization to cocaine; (2) lack of the characteristic Long-Term Synaptic Depression (LTD) response to cocaine in the core of the NAc; and (3) failure to upregulate some of the known molecular targets of CPEBs, whose expression is readily increased in response to cocaine in wild-type mice. Moreover, we found that in the DN-CPEB mice, the expression of FosB, a hallmark of drug addiction (Nestler et al., 2001; Nestler, 2008) that we here show to be a direct target of CPEBs molecules, is affected by acute administration of cocaine.

We conclude that CPEB1 and CPEB3 have important roles in both initiating and maintaining changes in gene expression after drug exposure by affecting not only known target mRNAs of CPEBs but also two newly identified CPEB target mRNAs: FosB and its spliced form ΔFosB, whose expression increases following chronic cocaine treatment (Nestler et al., 2001).

## Materials and Methods

### Animals

Male C57BL6/J mice (10- to 12-weeks-old; Jackson Laboratories, Bar Harbor, ME, USA) were used for all experiments. Mice were kept in clear plastic cages (29.2 × 19 × 12.7 cm, N10 cage, Ancare, Bellmore, NY, USA) in groups of five with *ad libitum* food (Prolab IsoPro RMH3000, PMI Nutrition International LLC., Brentwood, MO, USA) and water. Mice were kept at a 12 h day/night cycle. All behavioral experiments were carried out during the light phase. All animal procedures were executed following institutional guidelines. All mice were naive before all experimental procedures unless otherwise stated.

### Drug Treatment

Cocaine hydrochloride (Sigma, St. Louis, MO, USA; 10, or 30 mg/ kg) was dissolved in sterile saline and injected i.p. For molecular studies, cocaine was administered at a concentration of 30 mg/kg for 30 min and 24 h. For behavioral studies, the dosage of cocaine was decreased to 20 mg/kg for the motor sensitization assay and 10 mg/kg for the Conditional Place Preference task.

### Behavior

#### Open Field Studies

Locomotor activity was assessed using five Plexiglas open field boxes 43 times 43 cm^2^ (MED Associates, Georgia, VT, USA). Two sets of 16 pulse-modulated infrared photo beams were placed on opposite walls 2.5-cm apart to record x–y ambulatory movements. Activity chambers were computer-interfaced for data sampling at 100-ms resolution. Overall locomotor activity was quantified as the total distance traveled (cm). Mice were divided into two groups (*n* = 10 each): (i) saline i.p., (ii) cocaine i.p. Locomotor activity was measured by an automated video tracking system. Following 3 days of habituation in the open field area, on each day, mice were injected (i.p.) with 20 mg/kg of cocaine, and the locomotor activity (measured as total distance traveled) was recorded by an automated tracking system.

#### Conditioned Place Preference

C57BL6/J mice undergo conditioned place preference (CPP) testing as previously described (Levine et al., 2011). Briefly, on day 1, mice are allowed 30 min of free access to both sides of the place preference chamber (Med Associates) to determine initial preference. For 7 days, mice are conditioned (30 min) to saline on the initially preferred side, and the next day, to cocaine on the least-preferred side. On day 8, mice are again given free access to both sides of the chamber for 30 min to test their zone preference. The CPP is expressed as the difference in time spent in the cocaine-paired zone on day 1 and the time spent in the same compartment on day 8 after conditioning.

#### Electrophysiology

Experiments were performed on coronal sections of mouse brain slices prepared from C57/Bl6 mice 5–8 weeks old. The whole brain was placed in ice-cold artificial cerebrospinal fluid (ACSF) and a block of tissue containing the NAc was removed. Coronal sections (400 μm), from 0.8–1.4 mm before bregma, were cut with a vibratome and transferred to an interface chamber. Generally, two slices were obtained from each hemisphere. Slices were submerged and constantly perfused with ACSF at a rate of 2 ml/min and bubbled with 95% O_2_ and 5% CO_2_. The composition of ACSF was as follows (in mM): 124 NaCl, 1.2 MgSO_4_, 4 KCl, 1.0 NaH_2_PO_4_, 2 CaCL_2_, 26 NaHCO_3_, and 10 D-glucose. The temperature of the slices was maintained at 27°C. Experiments were started 2–3 h after the slices were dissected. Extracellular recordings were made using ACSF-filled glass electrodes. Stimuli were delivered at a rate of one per minute (0.017 Hz, 0.05 ms pulse duration) through concentric bipolar stainless steel electrodes (25 μM wire diameter, CBBRC75, FHC, Bowdoinham, ME, USA). Both recording and stimulating electrodes were positioned in the NAc immediately adjacent to the anterior commissure (core), with a distance of approximately 400–500 μm between the stimulating and recording electrodes. Local stimulation elicits a field potential containing two negative components (N1 and N2). N1 represents the presynaptic fiber volley and N2 represents the glutamatergic excitatory synaptic transmission in the Nac (Nicola et al., 1996; Hoffman et al., 2003; Winder, 2003).

### Molecular Biology

#### Antibodies and Immunoblotting

Mice were treated with cocaine either acutely (30 min) or chronically 24 h. Striata were dissected in PBS on ice and homogenized in 50 mM Tris·HCl (pH 7.5), 1 mM PMSF, and protease inhibitors (Roche tablets). Protein was measured using a Nanodrop spectrophotometer; 30–40 μg of protein was used for the detection of both CPEB1-3 and their targets’ expression and separated by 8% SDS-PAGE. After electrophoresis, gels were transferred to PVDF membranes (Protran, Schleicher and Schuell) and probed with the respective antibodies overnight at 4°C. Blots were incubated with an anti-rabbit IgG-HRP as the secondary antibody (1:1,000; Sigma, St. Louis, MO, USA) for 1 h at room temperature and quantified by using ECL (Amersham Pharmacia). To verify the accuracy of sample loading, selected blots were re-probed with a monoclonal antibody to β-tubulin. Relative optical density for the obtained bands was determined by using the densitometry program (ImageJ) from five independent sample pairs. The optical density of components corresponding to the CPEB proteins was normalized with the optical density from β-tubulin-specific bands for each animal.

#### mRNA Isolation and cDNA Preparation From Mouse Brain and qPCR Analysis

RNA was extracted from the striata of mice that had been injected with cocaine with Trizol reagent (Invitrogen, Waltham, MA, USA) and precipitated with isopropanol. mRNA was reverse-transcribed by using a SuperScript III First-Strand Synthesis kit (Invitrogen, Waltham, MA, USA). The amount of cDNA was quantified using real-time PCR.

### Statistical Analysis

The differences between the two groups were evaluated using the Student’s *t*-test. The differences between more than two groups were evaluated by the one-way ANOVA test. *Post hoc* multiple comparisons were performed using Tuckey’s test. The data normality was also tested.

## Results

### Cocaine Treatment Increases the Expression of CPEB1 and CPEB3 in the Striatum of Mice

**Figure 1:**
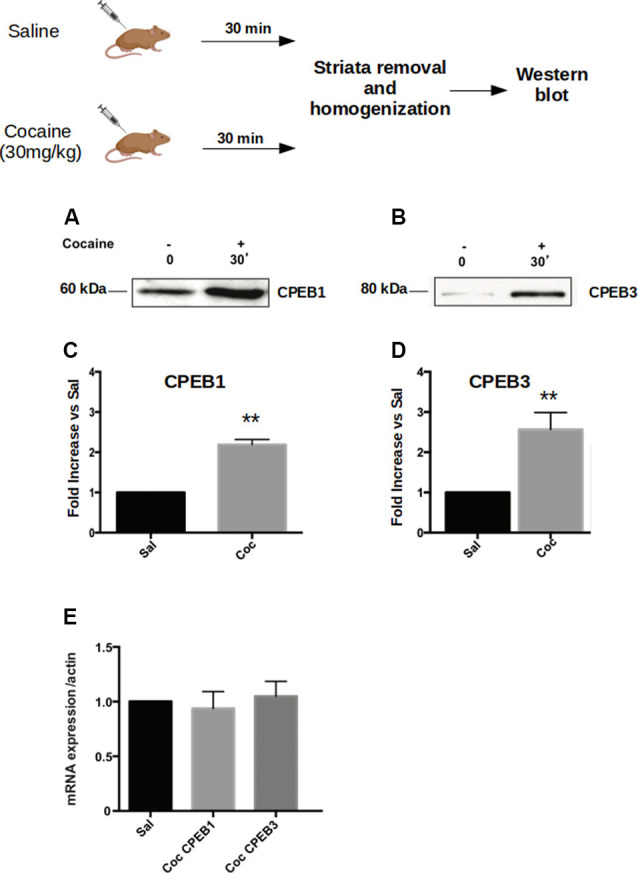
Cocaine administration induces Cytoplasmic Polyadenylation Element-Binding (CPEB3) upregulation in the nucleus accumbens (NAc). Striata of mice (*n* = 4) injected with 30 mg/kg of cocaine were analyzed 30 min after cocaine administration in a western blot assay. We found that the expression of CPEB1 and CPEB3 proteins increased considerably following an acute cocaine injection (**A–D**, *p* < 0.01). The increase in CPEB3 protein expression 30 min after an acute cocaine treatment is not transcriptionally dependent but is translationally driven. RT-PCR experiments revealed no significant differences in CPEB genes upregulation 30 min following cocaine exposure **(E)**. **p < 0.01.

Addictive behavior can be viewed as a pathological usurpation of the neural mechanisms of learning and memory, mechanisms that, under normal circumstances, serve to shape survival behaviors (Nestler, 2002; Chao and Nestler, 2004; Kelley, 2004; Hyman, 2005). To study this potential molecular parallel between addiction and long-term memory in the context of CPEB proteins, we first asked whether cocaine modulates the expression of the four CPEB family members, as does the formation of spatial memories (Fioriti et al., 2015). To test this idea, we injected C57BL/6J mice intraperitoneal (i.p.) with 30 mg/Kg of cocaine and sacrificed them 30 min after treatment. We then dissected the ventral striatum and homogenized it to analyze the expression of CPEBs proteins by western blotting. We found that, following acute cocaine injections, the protein levels of CPEB1, CPEB3, and CPEB4 increased (Figures 1A–D), while CPEB2 was not significantly upregulated (Supplementary Figure S1A). The upregulation of proteins 30 min after exposure to a stimulus is usually not dependent on changes at the transcriptional level but rather is translationally driven. We tested this idea using real-time PCR and asked whether the transcription of CPEB genes is altered by exposure to cocaine. We found that the mRNAs of the CPEB isoforms were not upregulated 30 min following cocaine exposure, suggesting that, at early time points cocaine indeed regulates the translation of CPEB1, CPEB3, and CPEB4 (Figure 1E). We noted, however, that two hours after cocaine treatment the levels of CPEB3 and CPEB1 mRNAs were higher than 30 min after cocaine treatment, with a corresponding more robust increase in the protein levels (Supplementary Figure S1B; *p* = 0.0043, *t*-test for CPEB3; data not shown for CPEB1).

### Cocaine Administration Results in the Upregulation of Target mRNAs of CPEB1 and CPEB3

Next, we investigated whether the increase of CPEB1, CPEB3 and CPEB4 protein expression 30 min after cocaine exposure (Figure 1), parallels an enhanced translation of their known mRNA targets. Toward this end, we injected C57BL/6J mice with 30 mg/Kg of cocaine i.p. and removed the ventral striatum after 30 min. Homogenates from the bilateral striatum were then used to analyze, by western blotting, the expression of some protein known targets of CPEB1, CPEB3, and CPEB4 (Huang et al., 2006; Pavlopoulos et al., 2011; Fioriti et al., 2015). We found that cocaine upregulates GluR1, tPa, beta-catenin, and CamKII, suggesting that enhanced protein levels of CPEB1 and CPEB3 induce an increased translation of their targets (Figure 5A). To investigate whether cocaine also modifies the activity of CPEB4, we tested the expression of its specific target Hif1. In contrast to CPEB1 and CPEB3, we found that cocaine does not induce upregulation of Hif1 in the striatum of treated mice (Supplementary Figure S1C), suggesting that the action of cocaine is quite specific. Cocaine does not increase the expression of all CPEB targets.

**Figure 2:**
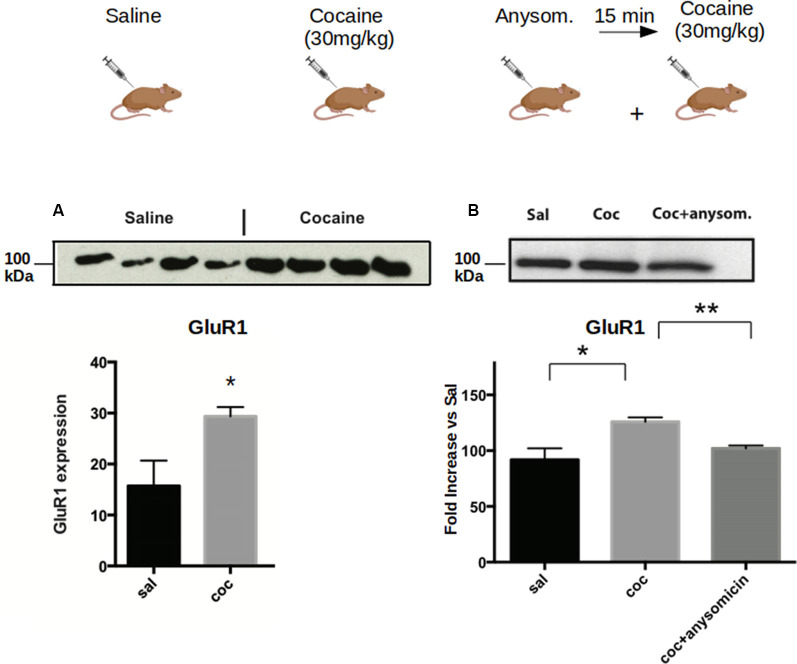
Cocaine induces increased expression of CPEB1 and CPEB3 that is dependent on translation. Striata from mice (*n* = 4) treated with acute cocaine injections were homogenized and used to analyze the expression of GLUR1, a shared target of CPEB1 and CPEB3. **(A)** GluR1 is upregulated following acute cocaine injection (*p* = 0.0001, Student’s *t*-test. **(B)**. The increased expression of the protein is not observed when mice have been previously injected with the translation inhibitor anisomycin (*p* = 0.86), suggesting that the cocaine-induced protein expression is translationally dependent. *p < 0.05, **p < 0.01.

**Figure 3:**
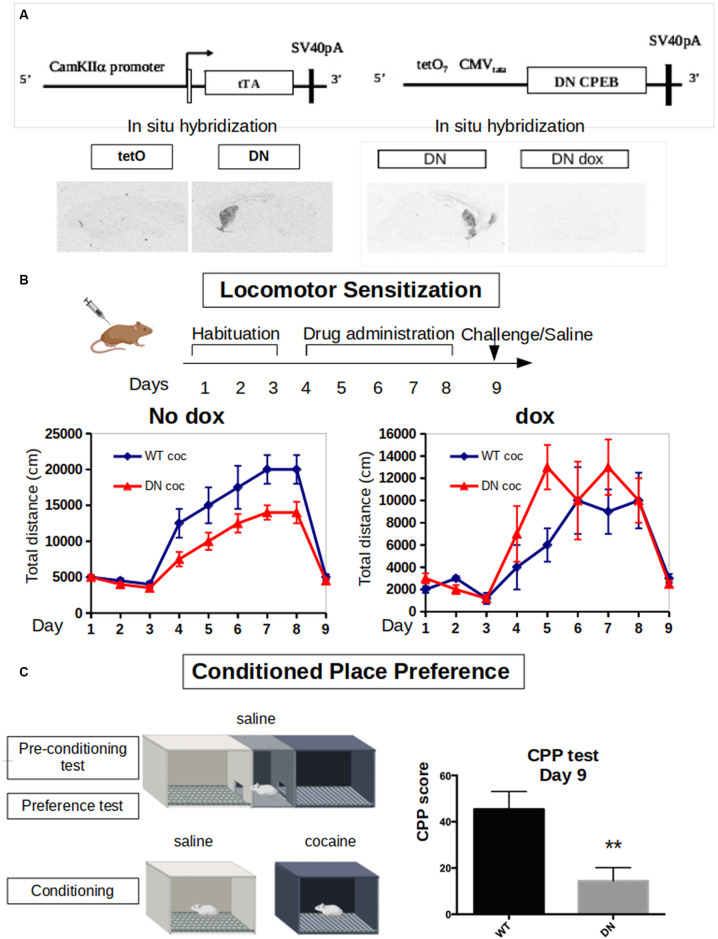
Dominant-negative DN-CPEB mice exhibit a deficit in both locomotor sensitization and Conditioned Place Preference (CPP) after cocaine treatment. **(A)** Schematic of the tetO and tTA transgenes of the DN-CPEB mice. *In situ* hybridization shows the expression of the transgene in the absence of doxycycline. **(B)** DN-CPEB mice were tested in a locomotor sensitization behavioral task. After 3 days of habituation in which both DN-CPEB and wild type (WT) mice, 10 per group, were shown to have similar baseline locomotion, mice were injected with cocaine 30 mg/kg for five consecutive days and their movement was recorded. DN-CPEB mice showed a considerable decrease in motor sensitization when compared to WT mice (*F* = 18.137; ANOVA, *p* < 0.0001). As control-CPEB mice were exposed to doxycycline (200 mg/kg) to prevent the expression of the TetO transgene. After 3 days of habituation in which both DN-CPEB dox and WT mice, 10 per group, were shown to have similar baseline locomotion, mice were injected with cocaine 30 mg/kg for five consecutive days and their movement was recorded. DN-CPEBdox mice showed no decrease in motor sensitization when compared to WT mice. **(C)** CPP was performed with the DN-CPEB and WT mice. After 4 days of cocaine pairing for both the DN-CPEB and WT animals, on the test day, the DN-CPEB mice spent less time in the cocaine-paired box compared to the WT mice (two-tailed *t*-test, *p* = 0.004), suggesting a potential role of CPEB1 and 3 in the rewarding properties of cocaine. **p < 0.01.

**Figure 4:**
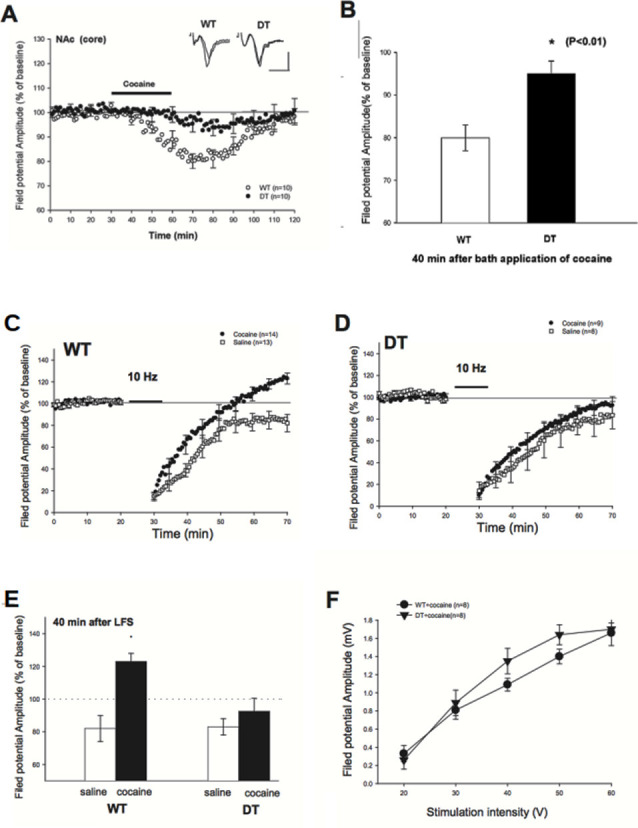
Reduced depression of field potential at baseline and Long-Term Synaptic Depression (LTD) blockade impairment in cocaine-treated DN-CPEB mice. **(A)** Brain slices from WT and DN-CPEB were used to record the extracellular field potential from the core of NAc in the presence or not of cocaine (30 μM), directly applied to the bath perfusion. **(B)** Quantification of the field potential amplitude at 40 min after cocaine application. DN-CPEB mice have a significantly different response compared to WT (*p* < 0.01). WT **(C,D)** DN CPEB mice (DT = double transgenic) slices were used to assess LTD in the excitatory synaptic transmission of NAc elicited by low frequency (10 Hz) stimulation. **(E)** Graphical representation of the quantification of the responses recorded in **(C,D)**. **(F)** Input/output curve of WT and DN-CPEB mice following cocaine administration as a function of increased stimulation intensity. *p < 0.05.

**Figure 5:**
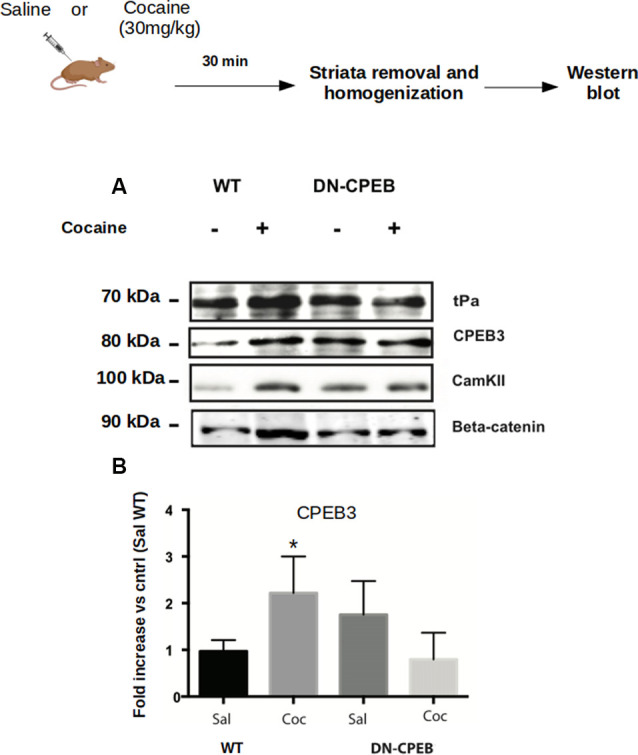
CPEB1 and CPEB3 target expression are impaired in DN-CPEBs mice. **(A)** Homogenates from WT and DN-CPEB mice (*n* = 4) treated with acute cocaine (30 mg/kg) were probed for the known target of CPEB1 and CPEB3. **(B)** Cocaine-induced CPEB1 and CPEB3 targets expression increase found in WT (*p* < 0.05) mice is abolished in DN-CPEB mice. **(B)** Quantification of CPEB3 in WT and DN-CPEB mice (Saline vs. Cocaine *p > 0.05). *p < 0.05.

To understand whether the increase in the protein levels of the targets of CPEB1 and CPEB3 was due to enhanced translation, we pre-treated the mice with anisomycin, an inhibitor of protein synthesis, and checked the expression of GluR1, a known target of both CPEB1 and CPEB3. We found that the expression of GluR1 was not increased following anisomycin treatment (Figure 2A), suggesting that the increased expression of the GluR1 protein is mediated by enhanced translation of existing mRNAs (Figure 2B).

### After Cocaine Treatment, Dominant-Negative DN-CPEB Mice Exhibit a Deficit in both Locomotor Sensitization and Conditioned Place Preference (CPP)

Considering the effects of cocaine in increasing the expression of both CPEB1 and CPEB3 and their targets, we next took advantage of a DN-CPEB mouse created in our laboratory. These transgenic mice express, under the control of a CamKII promoter, a dominant-negative form of CPEB1 affecting both CPEB1 and CPEB3. The CPEB1 coding region of the mutated mouse was placed under the control of the tet Operator (TetO) and a CaMKIIα tTA transgene (Mayford et al., 1996). In mice that were transgenic both for TetO DN-CPEB and CaMKII-tTA (DN-CPEB mice), we achieved forebrain-restricted expression in neurons (Figure 3A). We have therefore used these mice to understand the effects of cocaine administration in the absence of functional CPEB1 and CPEB3, at the behavioral, neurophysiological, and biochemical levels.

We first tested DN-CPEB mice in a locomotor sensitization behavioral paradigm where repeated exposure to drugs of abuse results in a progressive and long-lasting enhancement of the locomotor response, a phenomenon termed locomotor sensitization (Robinson and Berridge, 1993). Although the existence of sensitization in humans is disputed, locomotor sensitization in rodents has been proposed to parallel to certain aspects of drug addiction, such as compulsive drug-seeking behavior (Robinson and Berridge, 1993; Vanderschuren and Kalivas, 2000; Vezina and Leyton, 2009; Valjent et al., 2010). During the 3 days of habituation, where all mice were injected with saline and placed in the open field, the DN-CPEB mice did not exhibit any baseline motor deficit and showed a typical habituation pattern of locomotion.

We next treated 10 DN-CPEB and 10 wild type (WT) mice with daily i.p. injections using 20 mg/kg of cocaine for six consecutive days and recorded their locomotion in the open field. To obtain control data, another 10 DN-CPEB and 10 WT mice were treated with saline and their locomotion was recorded. DN-CPEB mice treated with cocaine demonstrated a significant decrease in motor sensitization when compared to WT mice (Figure 3B, *F*-value = 18.137; ANOVA, *p* < 0.0001). We then challenged the animals 7 days after the last cocaine injection and tested their locomotor response after saline only. We found that both genotypes responded in a similar way to saline administration 7 days after the last cocaine and saline administrations, confirming that the effect was specific to cocaine (Figure 3B).

As an additional control, we repeated the experiment but this time in the presence of doxycycline in the food pellets of the mice. Under this condition, the transgene is not expressed and the animals did not show any abnormality, thus confirming that the robust motor sensitization phenotype is dependent on the expression of the DN-CPEB gene (Supplementary Figure S2B, *p* = 0.82340).

Environmental cues associated with drug use elicit drug craving and a conditioned preference for the area where the drugs were consumed. We, therefore, subjected the DN-CPEB mice to a CPP behavioral task. On day 1, WT and DN-CPEB mice were given free access to two chambers. Then, for the next 8 days, mice were alternatively injected with saline or cocaine, and access to the other chamber was blocked. On the last day of the experiment, mice were allowed to freely explore both chambers and the amount of time spent in the cocaine-paired chamber compared to the saline one was analyzed.

The DN-CPEB animals exhibited a significant decrease in the time they spent in the cocaine-paired box compared to the WT littermate controls (Figure 3C; *t*-Student two-tailed *p* = 0.004). This suggests that mice lacking functional CPEB1 and CPEB3 show a decrease in the reward value of cocaine and implies that these translational regulators play an important role in determining the effects of cocaine on animal behavior.

### Reduced Depression of Field Potential at Baseline and Impairment of LTD Blockade in Cocaine-Treated DN-CPEB Mice

Electrophysiological experiments have found that drugs of abuse cause a decrease in the firing of NAc neurons (Wise, 1998) and that such decrease is associated with behavioral effects, such as enhanced locomotor activity in the sensitization paradigm (Pennartz et al., 1994). Also, there is a decrease in potentiation in brain slices after low frequency (10–13 Hz) stimulation that normally lead to Long-Term Synaptic Depression (LTD; Fourgeaud et al., 2004; Martin et al., 2006; Moussawi et al., 2009).

To examine whether DN-CPEB mice have a different electrophysiological response to cocaine, we first examined the depressive effects of cocaine in the NAc of WT and DN-CPEB mice after bath-applying the cocaine directly to the brain slices (Nicola et al., 1996). We recorded extracellular field potentials from the core of NAc. In WT mice, the amplitude of the field potential started to decline 10 min after the perfusion of cocaine (30 μM) and reached a maximum decline in about 40 min after the application of cocaine (80 ± 3% of baseline, *n* = 10). By contrast, the application of cocaine produced only a weak depression of field potential in DN-CPEB mice (95 ± 3%, *n* = 10, Figure 4A). This result indicated that there is a significant difference in the cocaine-induced synaptic depression in the core of NAc between WT and DN-CPEB1-3 mice (Figure 4B, *P* < 0.01).

Next, we asked whether an acute injection of cocaine affects LTD in the NAc and whether there is any difference between WT and DN-CPEB mice in the cocaine-induced LTD changes. We found that a brief exposure (10 min) to cocaine abolished the late phase of LTD in WT mice: in saline-treated WT mice, the field potential was decreased to 82 ± 5% (*n* = 13) of baseline level 40 min after the low frequency (10 Hz) stimulation. By contrast, the field potential was increased to123 ± 5% (*n* = 14) of baseline in the cocaine treated WT mice, which is significantly different from the controls (Figure 4C, *P* < 0.01). However, the blockade of LTD could not be observed in the DN-CPEB mice. The low-frequency stimulation produced a weak synaptic depression (92 ± 8%, *n* = 9) in the cocaine-treated DN-CPEB mice, which was not significantly different from the control (83 ± 5%, *n* = 8, *P* > 0.05, Figure 4D). Moreover, there were no significant differences in the input-output curve between WT and DN-CPEB mice (Figures 4E,F). Together, these results suggest that, after cocaine exposure, CPEB1 and CPEB3 control the blockade of LTD that is relevant for the neural adaptions necessary for the development of addictive behavior.

### The Translation of CPEBs Target mRNAs Is Reduced in DN-CPEB Transgenic Mice

To examine the behavioral and neurophysiological phenotypes observed at the molecular level in the DN-CPEB mice, we analyzed the activity-dependent expression of some known targets of CPEB1 and CPEB3.

We analyzed the expression of targets specific for CPEB1 or CPEB3, such as CamKII, tPA, CPEB3, beta-catenin, and GluR1 after acute injections of 30 mg/Kg of cocaine in both WT and DN-CPEB transgenic mice. Striata were removed 30 min after injections. Acute treatment with cocaine resulted in increased expression of CPEB1 specific targets in WT mice, but failed to induce a similar upregulation of protein expression in the DN-CPEB mice, suggesting that the DN-CPEB abolishes protein translation of key molecules implicated in these forms of synaptic plasticity (Figure 5).

### FosB Is a CPEB Target Whose Expression Is Misregulated in the Striatum of DN-CPEB Mice Following Cocaine Injections

Cocaine injections in WT mice trigger addictive-like behaviors that are associated with the upregulation of FosB (Nestler, 2014). To examine the phenotypic impact of CPEB1 and CPEB3 function in mice in response to cocaine administration, we asked whether FosB mRNA, whose expression contributes to the addictive phenotype, could be a target of CPEBs molecules. As a first step, we carried out *in silico* analysis and found that FosB contains a CPE element at the 3′UTR of its mRNA and thus could potentially be bound by both CPEB1 and CPEB3. It has been proposed (Huang et al., 2006) that CPEB3 can also recognize stem-loops secondary structures in the 3′UTR of target mRNAs. We used two different software (RNA fold and mRNA) to analyze the 3′UTR of FosB and found that the CPE element is located within a region that is predicted to fold in a stem-loop with high probability (Supplementary Figure S2), thus suggesting that both canonical CPE sequence recognition and stem-loop binding might contribute to the regulation of FosB 3′UTR binding by CPEB1 and CPEB3, respectively.

**Figure 6:**
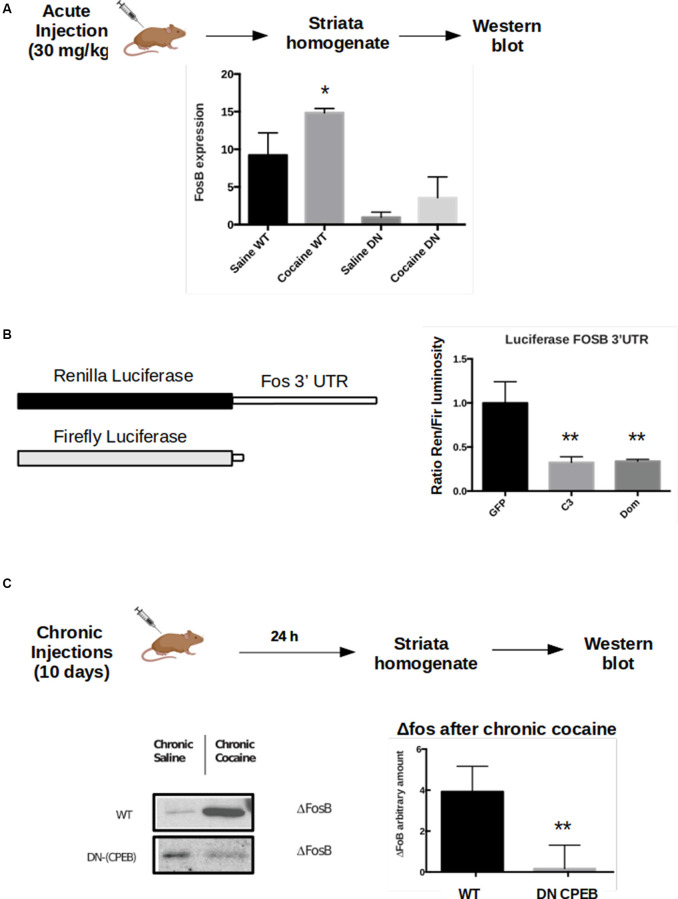
FosB is a CPEB3 target whose expression in the striatum is misregulated following cocaine injections. WT and DN-CPEB mice (*n* = 5) treated with acute cocaine. **(A)** FosB expression increases in WT but not in the DN-CPEB suggesting that FosB could be a direct target of CPEB 1 and 3. **(B)** Dual-Luciferase assay on HEK cells co-transfected with FosB 3′UTR Renilla plasmid and Firefly luciferase, in the presence of CPEB3 or DN-CPEB. The presence of CPEBs induces significantly less translation of the Renilla Luciferase gene, a shown in the change of renilla/Firefly ratio. **(C)** ΔFosB, a spliced form of FosB, is highly expressed in the NAc after chronic cocaine i.p. injections. ΔFosB expression increase following chronic cocaine injections in WT mice but not in DN-CPEB transgenic (*n* = 5, *p* < 0.01, Student’s *t*-test). *p < 0.05, **p < 0.01.

To test whether FosB expression is affected in the DN-CPEB animals, we injected WT and DN-CPEB mice with cocaine i.p. and found that 15 min after cocaine injection, FosB protein expression increases in WT but not in the DN-CPEB mice (Figure 6A). This result suggests that FosB could be a direct target of the two CPEB family members since its protein induction is compromised in DN-CPEB mice after cocaine exposure. To further investigate this idea, we performed a luciferase assay to analyze, *in vitro*, the expression of FosB in the presence or absence of DN-CPEB. We cloned the 3′UTR of FosB in a Renilla luciferase plasmid and transfected HEK 293 cells together with a plasmid encoding for the DN-CPEB gene. We found that when we co-transfected HEK 293 cells with plasmids encoding for FosB 3′UTR Renilla and the DN-CPEB gene, we obtained significantly less translation of the Luciferase gene. This suggests that the FosB mRNA is a CPEB direct target and is repressed in the basal state (Figure 6B), as is the case for all other known CPEBs targets. As we mentioned in the introduction, the DN-CPEB protein is a CPEB1 protein deleted of the N-terminal regulatory region that can still bind mRNA targets and can, therefore, repress their translation. To determine whether FosB is a target of CPEB3 as well as of CPEB1, we co-expressed, in HEK 293 cells, the FosB 3′UTR Renilla plasmid together with a plasmid encoding CPEB3. We found that CPEB3 overexpression also decreases the translation of the luciferase gene, suggesting that FosB is a target of both CPEB1 and CPEB3.

### ΔFosB Long-Term Expression Decreases in DN-CPEB Mice

Although FosB family members are induced rapidly, albeit transiently, in response to acute stimuli, one isoform, ΔFosB, is characterized by a long-term induction in response to repeated stimulations thanks to its stability. ΔFosB prolonged induction, within the NAc, promotes reward and motivation and serves as a mechanism of drug sensitization (Nestler, 2008, 2015). ΔFosB expression increases following repeated cocaine administration (and administration of other drugs of abuse) and is stable over several weeks after the last exposure. Since both locomotor sensitization and CPP behavioral tasks require chronic injections of cocaine, we asked whether ΔFosB expression also changes in DN-CPEB mice. After treating the transgenic mice with cocaine for 10 days, we found that ΔFosB was significantly reduced in DN-CPEB mice compared to WT mice (Figures 6C,D, *P* < 0.0001), suggesting that CPEB1 and CPEB3 function as translational regulators of ΔFosB mRNA expression and that they might help to maintain an addictive-like phenotypic behavior over-time, similar to their role in the maintenance of long term spatial memories (Fioriti et al., 2015).

## Discussion

Our results indicate that CPEB1 and CPEB3, previously known for their role as translational regulators of targets important in synaptic plasticity and long-term spatial memory maintenance in the hippocampus (Mendez and Richter, 2001; Fioriti et al., 2015), also have an important role in addiction by shaping the neuronal adaptation to cocaine administration in the striatum.

Our studies of transgenic mice expressing a dominant-negative form of CPEB1 that also affectsCPEB3 function illustrates that these CPEBs have roles as translation regulators of CPE-bearing mRNA targets necessary for the formation of both cocaine-induced sensitization and conditional place preference (CPP). Following cocaine administration, mice lacking functional CPEB1 and CPEB3 exhibited characteristically impaired addiction-like behavior, such as reduced sensitization to cocaine, absence of the characteristic LTD response to cocaine in the core of the NAc, and failure in upregulating known molecular targets of CPEBs, whose expression is normally increased in response to cocaine in wild-type mice. Interestingly, we found that both FosB and ΔFosB are the direct targets of CPEB1 and 3 and that their translation is affected in DN-CPEB transgenic mice. An important aspect of our studies is that the dominant-negative mutant of CPEB1 also affects CPEB3 since the translation of CPEB3 is regulated by CPEB1. Therefore DN-CPEB mice have impaired CPEB1 and CPEB3-mediate translation. To better elucidate whether the defects observed in the DN-CPEB mice are mainly driven by CPEB1 or by CPEB3 more studies will be necessary. For example, one could restore selectively the function of CPEB3 in the DN-CPEB mice, using a viral expression, or with a more direct approach, test the phenotype of CPEB3 knockout mice exposed to cocaine. These future studies will provide a deeper understanding of the molecular mechanisms underlying addictive behavior.

The clear overlap between the molecular processes involved in normal learning and memory and the pharmacological actions of drugs of abuse has led many scientists to hypothesize that addiction develops, at least in part, because of aberrant learning and memory (White, 1996; Jentsch and Taylor, 1999; Everitt et al., 2001; Kilts, 2001; Kelley, 2004; Hyman et al., 2006; Thomas et al., 2008). Our studies provide the first direct support for this hypothesis.

A common feature of drugs of abuse is that, despite different mechanisms of action, they all cause an increase in the release of dopamine in the striatum (Di Chiara, 1999; Torregrossa and Kalivas, 2008), affecting the ability to learn about a new event happening in the environment (Shultz and Dunbar, 2010). This effect is magnified each time the drug is consumed. Chronic drug exposure not only produces long-lasting changes in behaviors produced by learning and memory, but it also produces enduring plastic changes in the molecular signaling pathway of circuits that underlie learning and memory. Specifically, these changes occur in three common molecular pathways: (1) cAMP/PKA/CREB; (2) ΔFosB/Cdk5; and (3) BDNF/ERK signaling cascades.

For many years, studies of the molecular mechanism of drugs of abuse have focused heavily on transcriptional and epigenetic regulatory mechanisms. ΔFosB, a molecule that is upregulated by the chronic administration of nearly all drugs of abuse, is considered to be a molecular switch to addiction, but also, to be one of the molecular determinants of the persistence of drug addiction. In both cultured cells *in vitro* and the NAc *in vivo*, the 35–37 kD ΔFosB isoforms have been found to accumulate after chronic use of cocaine (and other drugs of abuse) due to their extraordinarily long half-lives (Nestler, 2008). Because of its stability, the ΔFosB protein persists in neurons for several weeks after cessation of drug exposure. This stability is due to two factors: (1) the absence from ΔFosB of two degron domains that are present at the C-terminus of full-length FosB and target the proteins for rapid degradation; and (2) ΔFosB phosphorylation at its N-terminus (Ser27) by CaMKII (Ulery et al., 2006). Nevertheless, ΔFosB expression inevitably drops hours after the last drug administration and thus cannot fully explain lifelong addictive behavior.

Here, we find a role for the translational regulators CPEB1–3 in mediating the effect of cocaine in the striatum and the NAc. Except for mTORC1, which indirectly controls synaptic protein translation (Hoeffer and Klann, 2010) in various learning and memory paradigms and drug addiction as well (Costa-Mattioli et al., 2009; Hoeffer and Klann, 2010; Neasta et al., 2014), CPEB1–3 are one of the few examples of translational regulators involved in drug addiction dependent plasticity.

Interestingly, we find that FosB is a direct target of both CPEB1 and CPEB3 and its translation depends upon the proper functioning of CPEBs, since, in our DN-CPEB mouse, the expression of FosB and its spliced form ΔFosB decreases following acute and chronic cocaine administration, respectively (Nestler, 2008).

In previous work, our laboratory and others have found that the translational regulator CPEB is an important component in the maintenance of learned memory in Aplysia, Drosophila, and mice (Si et al., 2003a; Majumdar et al., 2012; Fioriti et al., 2015) as a consequence of their prion-like properties (Si et al., 2003b, 2010; Stephan et al., 2015). A possible mechanism to explain this long-term memory/addiction-like persistence could be the combined role of the transcriptional regulators FosB/ΔFosB and the translational regulators CPEB1 and CPEB3 in establishing a long-term feedback loop in the presence of chronic exposure to cocaine that helps maintain the synaptic plasticity associated with drug addiction.

Although some studies have found that the persistent expression of ΔFosB is due to the enhanced stability of the ΔFosB protein, rather than increased mRNA translation as described above, we here raise the possibility that a mechanism such as CPEBs-mediated enhanced translation could prolong the stability of the ΔFosB protein over the long run and that the two mechanisms, stability, and increased translation, could have a positive feedback upon drug stimuli.

Also, we find that, after acute and chronic cocaine administration, FosB and ΔFosB expressions are regulated at the translational level by CPEB1–3, which are implicated in the maintenance of long-term memory, especially in the case of CPEB3 that has prion-like proteins characteristics (Si et al., 2003a,b; Stephan et al., 2015).

Although more studies are required to understand in detail the relationship between the transcriptional regulator FosB/ΔFosB and its translational regulators, CPEB1 and CPEB3, the discovery of a bilateral interplay between the two molecules might shed light into the important relationship between transcription and translation in mediating the long-term effects of cocaine.

## Data Availability Statement

The raw data supporting the conclusions of this article will be made available by the authors, without undue reservation.

## Ethics Statement

The animal study was reviewed and approved by IACUC, Columbia University.

## Author Contributions

BD, LC, AL, YH, and LF designed the experiments. BD, LC, AL, YH, AS, and DM performed the experiments and analyzed the data. MT generated the DN-CPEB mice. AL, LF, DK, and EK supervised the work. BD, LC, AL, YH, DK, EK, and LF wrote the manuscript.

## Conflict of Interest

The authors declare that the research was conducted in the absence of any commercial or financial relationships that could be construed as a potential conflict of interest.

## Supplementary Material

FIGURE S1 CPEB1 and CPEB3 targets are selectively upregulated by cocaine. **(A)** CPEB2 levels are not increased by cocaine injection. **(B)** CPEB4 levels are significantly increased after cocaine injections (*n* = 4, *p* < 0.01), but **(C)** CPEB4 targets are not, either in WT or DNCPEB mice, suggesting that CPEB4 is not under CPEB1 translational control (ANOVA, not significant). **p* < 0.05, ***p* < 0.01.

FIGURE S2 Dominant-negative DN-CPEB mice express a mutant, truncated CPEB1 that can bind to FosB 3′UTR. **(A)** DN-CPEB mice specifically express the TetO transgene in both hippocampus and striatum as shown by immunoblot with anti-Flag antibody. **(B)** CPEB3 mRNA is upregulated after long-term exposure to cocaine. The increase in CPEB3 protein expression 2 h after an acute cocaine treatment is transcriptionally dependent. RT-PCR experiments reveal significant differences in CPEB genes upregulation 30 min following cocaine exposure (*n* = 4, *p* < 0.01, *t*-test). **(C)** The minimum free energy (MFE) structure of FosB 3′UTR is shown. The CPE element (UUUAU) recognized by CPEB1 is highlighted in pink. The CPE is also part of a stem-loop structure. The MFE structure was obtained with the software RNA fold (http://rna.tbi.univie.ac.at).
