# Supplementary figures and images for: Cytoplasmic Polyadenylation Element Binding Proteins CPEB1 and CPEB3 Regulate the Translation of FosB and Are Required for Maintaining Addiction-Like Behaviors Induced by Cocaine
Source: Front Cell Neurosci. 2020 Jul 9;14:207. doi: 10.3389/fncel.2020.00207 (PMC7365288; doi:10.3389/fncel.2020.00207)

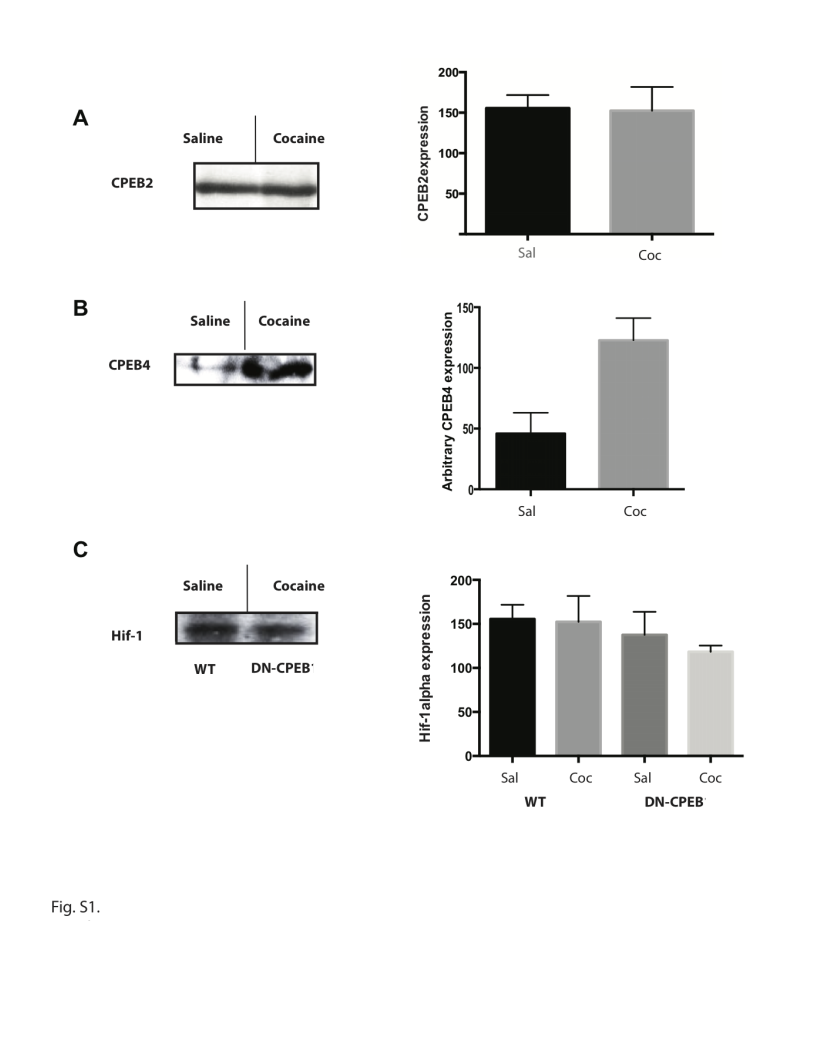

Supplement: FIGURE S1 — CPEB1 and CPEB3 targets are selectively upregulated by cocaine. (A) CPEB2 levels are not increased by cocaine injection. (B) CPEB4 levels are significantly increased after cocaine injections (n = 4, p < 0.01), but (C) CPEB4 targets are not, either in WT or DNCPEB mice, suggesting that CPEB4 is not under CPEB1 translational control (ANOVA, not significant). *p < 0.05, **p < 0.01. [file Image_1.TIF]

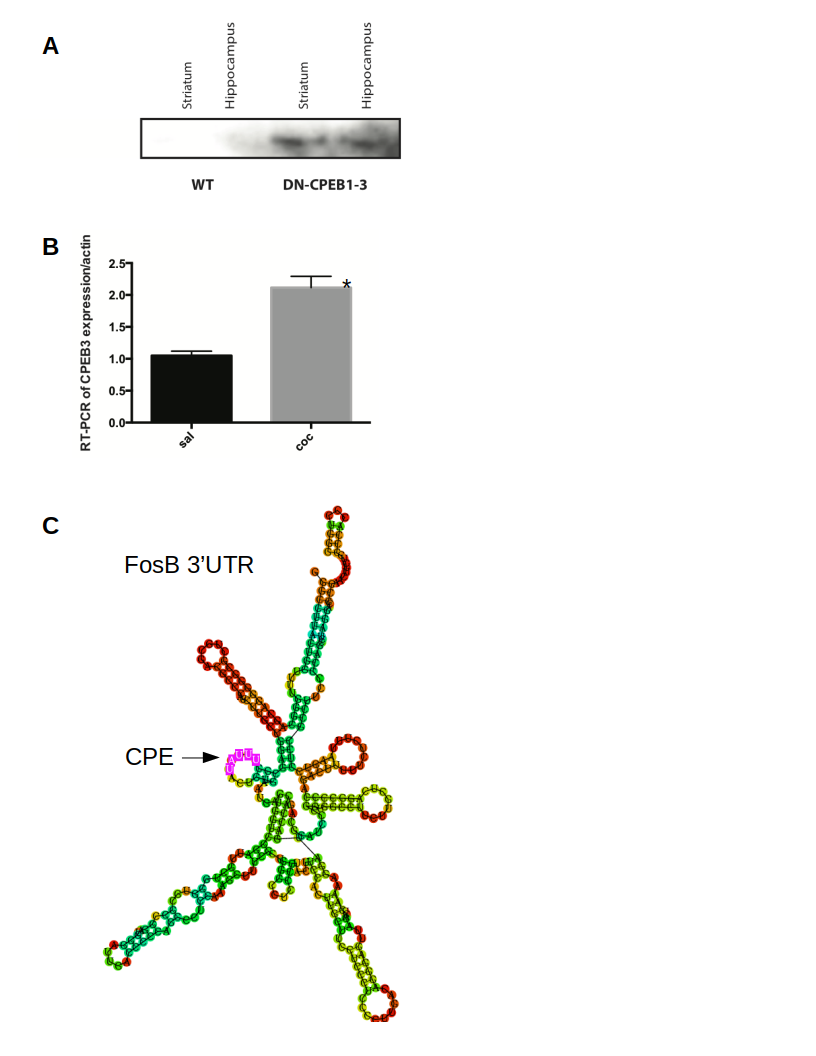

Supplement: FIGURE S2 — Dominant-negative DN-CPEB mice express a mutant, truncated CPEB1 that can bind to FosB 3′UTR. (A) DN-CPEB mice specifically express the TetO transgene in both hippocampus and striatum as shown by immunoblot with anti-Flag antibody. (B) CPEB3 mRNA is upregulated after long-term exposure to cocaine. The increase in CPEB3 protein expression 2 h after an acute cocaine treatment is transcriptionally dependent. RT-PCR experiments reveal significant differences in CPEB genes upregulation 30 min following cocaine exposure (n = 4, p < 0.01, t-test). (C) The minimum free energy (MFE) structure of FosB 3′UTR is shown. The CPE element (UUUAU) recognized by CPEB1 is highlighted in pink. The CPE is also part of a stem-loop structure. The MFE structure was obtained with the software RNA fold (http://rna.tbi.univie.ac.at). [file Image_2.TIF]
